# State Legislator Social Media Posts About the 988 Suicide and Crisis Lifeline

**DOI:** 10.1001/jamanetworkopen.2023.39845

**Published:** 2023-10-26

**Authors:** Jonathan Purtle, Michael Soltero, Margaret E. Crane, Anna-Michelle Marie McSorley, Molly Knapp, Christopher W. Drapeau

**Affiliations:** 1 Department of Public Health Policy and Management, School of Global Public Health, New York University, New York, New York; 2 Department of Psychiatry, New York Presbyterian-Weill Cornell Medicine, New York, New York; 3 Center for Anti-racism, Social Justice, and Public Health, School of Global Public Health, New York University, New York, New York; 4 988 Suicide and Crisis Lifeline, Vibrant Emotional Health, New York, New York; 5 Department of Health Policy and Management, Indiana University Richard M. Fairbanks School of Public Health, Indianapolis

## Abstract

This cross-sectional study examines the volume and content of state legislator social media posts about the 988 National Suicide and Crisis Lifeline.

## Introduction

On July 16, 2022, “988” became the new national dialing code for the National Suicide and Crisis Lifeline.^1^ The ease of the 3-digit dialing code and broadening of the Lifeline scope to include nonsuicidal mental health and substance use crises has potential to improve service access when rates of these issues are increasing.^2,3^ However, public awareness of 988 is low.^4^

Although 988 was created by federal law, states have broad discretion regarding implementation. A 2023 study^5^ documented significant between-state variation in legislative approaches to 988 implementation and magnitudes of increase in 988 call volume. Understanding state legislator social media posts about 988 may be informative because their posts can influence the knowledge and behaviors among their constituents (eg, 988 awareness and use).^6^ This descriptive study characterized variation in the volume and content of state legislator social posts about 988 by date, state, and political party.

## Methods

This cross-sectional study was exempted from review by the New York University institutional review board because it involves only publicly available information. Informed consent was not required because this was not human participants research. The report is presented in accordance with STROBE reporting guidelines.

Quorum, a comprehensive legislative database used in prior work,^6^ was used to identify social media (Facebook and Twitter, including retweets) posts mentioning 988 from the accounts of state legislators and Washington, District of Columbia, council members between January 1 and December 31, 2022 (approximately 6 months before and after 988 launch). The search string used is in the eMethods in Supplement 1. A coding instrument (eMethods in Supplement 1) was developed, and reliability was assessed by double coding a random 10% sample of posts. Interrater agreement was 93% or higher for all categories presented. The coded data set and additional tables are publicly available (Supplement 2).

The number of 988 posts per month was calculated and stratified by state and political party. To account for variability in the number of legislators in each state and size of each political party, state- and party-specific rates of 988 posts per 10 000 posts were calculated. Quorum was used to estimate the total number of posts by state and party each month. The percentage of 988 posts coded to each category was calculated; χ^2^ tests were used to assess the significance (*P* < .05) of differences in posts between Democrats and Republicans. Analyses were conducted in SPSS statistical software version 28 (IBM).

## Results

A total of 1000 state legislators posted 2041 social media posts mentioning 988 in 2022 (52.2% tweets and 47.8% Facebook posts). State-level post rates per 10 000 posts ranged from a high of 132.7 in California to 1.4 in West Virginia. In 11 states, fewer than 10 posts mentioned 988 in 2022.

Among these posts, three-quarters occurred in July (54.0%), when 988 launched, or September (21.6%), which is Suicide Prevention Awareness Month (Figure). The annual 988 post rate per 10 000 posts was 9.80 among Democrats (0.098% of all posts) and 6.83 among Republicans (0.068% of all posts) (*P* < .001).

**Figure.**
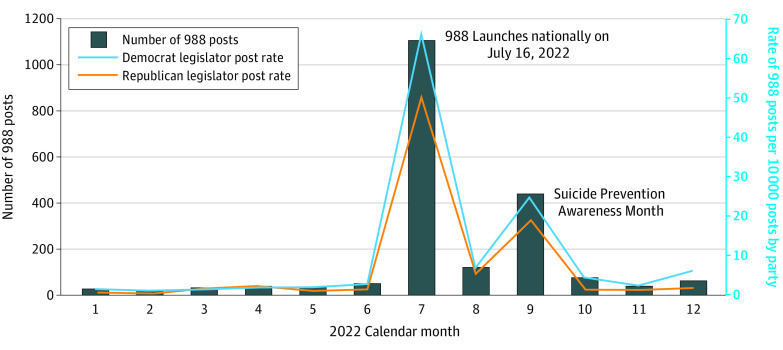
Volume of US State Legislator Social Media Posts Mentioning 988 The volume of social media posts mentioning the 988 National Suicide and Crisis Lifeline from January 1 to December 31, 2022, is presented, including the total number of posts per month and rate per 10 000 social media posts from Democrats and Republicans. Among approximately 1 592 500 total posts from Democrats, 1561 posts mentioned 988. Among approximately 682 700 total posts from Republicans, 466 posts mentioned 988.

Approximately half of legislator 988 posts (44.9%) were general information that did not encourage 988 use or mention government action related to 988 (Table). More than one-third of 988 posts (34.2%) explicitly encouraged use of 988, while 10.3% of posts mentioned government action related to 988 and 12.5% of posts mentioned that 988 could be used for substance use crises.

**Table.** Characteristics of US State Legislators Social Media Posts Mentioning 988

Thematic category	Example post from category	Posts, No. (%)			*P* value^a^
		All (n = 2041)	Democrats (n = 1561)	Republicans (n = 466)	
General news about 988^b^	“New 988 hotline is the 911 for mental health emergencies”	916 (44.9)	672 (43.0)	237 (50.9)	.03
Encourages use of 988 among people in distress or on behalf of loved ones	“For those in crisis, I encourage you to use the new 988 hotline, which will connect you with trained counselors.”	699 (34.2)	546 (35.0)	150 (32.3)	.33
Mentions legislative or government action related to 988	“Assembly Bill 988 creates a dedicated funding source to run Californian’s thirteen 988 call centers by attaching a small $0.08 a month telephone surcharge–that’s less than a dollar a year.”	211 (10.3)	160 (10.2)	49 (10.5)	.58
Mentions that 988 can be used for substance use issues^c^	“Struggling with thoughts of suicide or experiencing a mental health or substance use crisis? Call or text 988”	255 (12.5)	211 (13.5)	43 (9.2)	.06
Includes statistics about suicide, mental health, or substance use issues^c^	“988 may have saved more than 150,000 more lives in one month. Feels good, man. Calls to suicide prevention hotline increase by 45% after transition to new 988 number”	125 (6.1)	71 (4.5)	54 (11.6)	<.001

^a^ *P* values calculated from χ^2^ test.

^b^ Coded at this category only if the post did not encourage use of 988 and did not mention legislative or government action related to 988.

^c^ Could also be coded as general news about 988.

## Discussion

This cross-sectional study found that state legislators actively communicated about 988 on social media when 988 launched, with variability between states. However, such communication was not sustained over time and most posts did not explicitly encourage use of 988. Few 988 posts mentioned substance use, despite the intent of 988 to be inclusive of such crises. Limitations of this study include its descriptive and cross-sectional nature. Results suggest a potential need to increase and improve state legislator communication about 988, which may be associated with increased awareness about and use of the 988 Lifeline among the public.

## Notes

Supplement 1.
eMethods.

Supplement 2.
Data Sharing Statement
