# Supplementary material for: State Legislator Social Media Posts About the 988 Suicide and Crisis Lifeline
Source: JAMA Netw Open. 2023 Oct 26;6(10):e2339845. doi: 10.1001/jamanetworkopen.2023.39845 (PMC10603493; doi:10.1001/jamanetworkopen.2023.39845)
Supplement: Supplement 1. — eMethods. [file jamanetwopen-e2339845-s001.pdf]

Supplemental Online Content

Purtle J, Soltero M, Crane ME, McSorley AMM, Knapp M, Drapeau CW. State legislator social media posts about the 988 Suicide and Crisis Lifeline. *JAMA Netw Open*. 2023;6(10:e2339845.
doi:10.1001/jamanetworkopen.2023.39845

eMethods.

This supplemental material has been provided by the authors to give readers additional information about their work.

© 2023 Purtle J et al. *JAMA Network Open.*

eMethods.

Search String Used: ("988" OR "9-8-8") AND ("lifeline" OR "hotline" OR "suicide" OR "mental health" OR "crisis" OR "substance" OR "opioid")

Coding Instrument:

Q1 Coder name

- ○ (1)
- ○ (4)
- ○ (2)
- ○ (3)

Q2 Quorum ID (used to link to Quorum with information on post date, state, and political party).

____

Q8 Is the post a Retweet (content begins "RT")?

- ○ Yes (1)
- ○ No (2)

Q9 **General news:** Is the content a general news announcement about the launch of 988, **and does NOT** encourage help seeking, include news about legislative/government action, or funding/capacity challenges?

- ○ Yes (1)
- ○ No (2)

© 2023 Purtle J et al. *JAMA Network Open.*

Q10 **Encouraging self-help seeking:** Does the content include a call to action for people experiencing suicidality, mental distress, and/or substance use problems to call/chat/text 988 for themselves or on behalf of a loved one?

- ◯ Yes (1)
- ◯ No (2)

Q11 **News about legislative/government action:** Does the content mention of a bill being introduced about 988, a legislative hearing about 988, a government report about 988, or other legislative/government activity about 988?

- ◯ Yes (1)
- ◯ No (2)

Q13 **Substance use mention:** Does the content mention that 988 can be used for substance use crisis/concerns (i.e., not only suicidality or mental health concerns)?

- ◯ Yes (1)
- ◯ No (2)

Q14 **Statistics:** Does the content include statistics about suicide, mental health, or substance use?

- ◯ Yes (1)
- ◯ No (2)

© 2023 Purtle J et al. *JAMA Network Open.*
