# Supplementary material for: State Legislator Social Media Posts About the 988 Suicide and Crisis Lifeline
Source: JAMA Netw Open. 2023 Oct 26;6(10):e2339845. doi: 10.1001/jamanetworkopen.2023.39845 (PMC10603493; doi:10.1001/jamanetworkopen.2023.39845)
Supplement: Supplement 2. — Data Sharing Statement [file jamanetwopen-e2339845-s002.pdf]

Data Sharing Statement

Purtle. State Legislator Social Media Posts About the 988 Suicide and Crisis Lifeline. *JAMA Netw Open*. Published online October 26, 2023. doi:10.1001/jamanetworkopen.2023.39845

Data

Data available: Yes
Data types: Deidentified participant data **How to access data:** https://osf.io/mp9nr/**When available:** With publication

Supporting Documents

Document types: None

Additional Information

Who can access the data: Researchers whose proposed use of the data has been approved
Types of analyses: Secondary analysis
Mechanisms of data availability: OSF, at https://osf.io/mp9nr/
